# The laboratory of lung function in the follow-up of lung transplant recipients

**DOI:** 10.36416/1806-3756/e20250015

**Published:** 2025-03-18

**Authors:** José Alberto Neder, Denis E O’Donnell, Danilo C Berton

**Affiliations:** 1. Pulmonary Function Laboratory and Respiratory Investigation Unit, Division of Respirology, Kingston Health Science Center & Queen’s University, Kingston (ON) Canada.; 2. Unidade de Fisiologia Pulmonar, Hospital de Clínicas de Porto Alegre, Universidade Federal do Rio Grande do Sul, Porto Alegre (RS) Brasil.

## BACKGROUND

Lung transplantation significantly affects various domains of respiratory physiology. Some changes result from the procedure itself and any direct lung injury related to it. Post-transplantation chronic lung allograft dysfunction (CLAD) remains a major cause of morbidity and mortality.^1^ Therefore, lung transplant recipients should undergo regular pulmonary function tests (PFTs) as part of ongoing monitoring.^1^ ^,^ ^2^

## OVERVIEW

**Figure 1:**
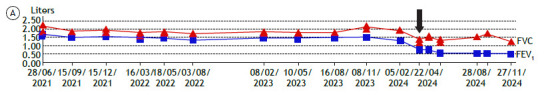

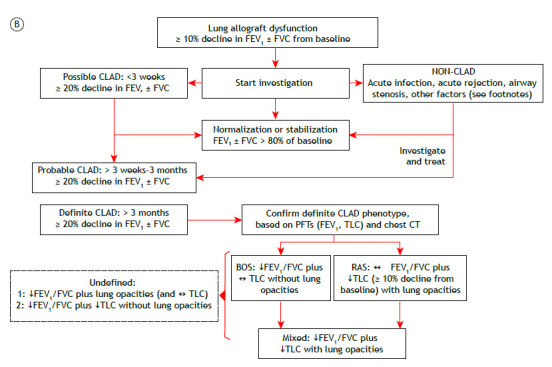
In A, serial spirometric measurements in a woman who underwent right lung transplantation because of severe COPD. The onset of chronic lung allograft dysfunction (CLAD) is indicated by a persistent drop (> 20%) in FVC and FEV_1_ (arrow) in the absence of new lung opacities. Given a greater reduction in the latter, FEV_1_/FVC turned abnormally low, signaling the presence of bronchiolitis obliterans syndrome (BOS). In B, a simplified approach to the diagnosis of CLAD in patients with lung allograft dysfunction.^1^ As outlined below, there are several modifiers that should be considered in the longitudinal interpretation of pulmonary function tests (PFTs) in this patient population.^1^ ↓: reduced; ↔: preserved; and RAS: restrictive allograft syndrome. A. Factors where recalculation/resetting of the FEV_1_ reference value may be valid (if FEV_1_ remains stable for at least 6 months): 1. decreasing lung function as a result of the normal aging process 2. surgical factors, including transplant lung resection, chest wall surgery, and phrenic nerve damage 3. mechanical factors, including persistent pleural effusion, persistent lung edema caused by significant kidney/heart/liver failure, myopathy, neuropathy, weight gain, and native lung hyperinflation after single-lung transplantation 4. localized infection with chronic scarring-abscess, empyema, and/or mycetoma B. Factors that cannot be differentiated easily from CLAD and do not ever allow recalculation/resetting of the FEV_1_ reference value: 1. any from (A) when there is not at least 6 months of stability 2. infiltration with tumor 3. infiltration of the allograft with proven disease recurrence from the underlying transplant indication (e.g., sarcoidosis and lymphangioleiomyomatosis) 4. drug or other induced pulmonary toxicity (e.g., sirolimus, methotrexate, amiodarone, and radiation therapy) 5. pulmonary arterial strictures or emboli 6. acute/subacute generalized infection 7. acute/subacute cellular or antibody-mediated rejection 8. acute/subacute effects of aspiration C. Failing to reach normal predicted lung function (i.e., low FEV_1_ reference value such that FEV_1_ is ≤ 80% of the recipient predicted value). This may occur when older donor lungs are implanted or when an intraoperative allograft reduction surgery/lobectomy is performed.

A 60-year-old woman underwent right lung transplantation because of severe emphysema. Despite mild acute lung rejection in the first post-transplant year, she remained largely asymptomatic with relatively preserved lung function over several years. However, she reported progressive exertional dyspnea after a severe lower respiratory tract infection six years later. Despite treatment optimization, there had been a persistent (> 3 months) decline in FEV_1_ (≥ 20%) relative to baseline (Figure 1A). The presence of obstruction (low FEV_1_/FVC) without restriction (preserved TLC) or new opacities on chest CT suggested the obstructive phenotype of CLAD (Figure 1B).^1^

Changes in PFTs after lung transplantation are influenced by the underlying lung disease of the recipient and whether the transplant is single or bilateral.^3^ ^,^ ^4^ Clinical interpretation of PFTs in recipients of single transplants is more complex because changes may reflect the progression of the underlying disease in the native lung. Most centers recommend (at least) spirometry once a month for the first post-transplant year and every 3-4 months subsequently. FVC and FEV_1_ usually improve over the first three months following surgery, and there is a slight further improvement up to 24 months after bilateral transplantation.^3^ ^,^ ^4^ The average of two maximal post-transplant FEV_1_ values obtained at least three weeks apart should be recorded as a baseline for monitoring allograft function.^1^ Supranormal FEV_1_/FVC might be seen, secondary to a restrictive thoracic cage due to the operative procedure and/or transplantation of large lungs, causing a mismatch between higher airflow capacity and thoracic cage volume. A persistent (> 2 days) decline of 10% in spirometric values has been reported to indicate either rejection or infection.^5^

CLΑD is an umbrella term describing a significant decline in lung function after lung transplantation in the absence of other identifiable causes. The most common manifestation of CLAD is bronchiolitis obliterans syndrome. However, up to 30% of patients with CLAD develop a restrictive phenotype. A diagnostic workup is provided in Figure 1B. More sensitive metrics of smaller airway dysfunction (such as low mid-expiratory flows and impulse oscillometry measurements) and/or air trapping (high functional residual capacity and RV) are not widely considered given the great variability and the lack of data from large studies examining this issue. However, persistent changes in these parameters and those reflecting impaired gas transfer (hemoglobin-corrected DL_CO_ and carbon monoxide transfer coefficient) might be relevant in individual subjects. 

## CLINICAL MESSAGE

PFTs are critical for monitoring allograft (dys)function; for early detection of rejection and infection; and for monitoring response to treatment. Careful clinical and imaging correlation is paramount. Close attention should be given to factors that can negatively impact lung function, such as weight gain, aging, comorbidities, and concurrent local or systemic pathological processes (Figure 1, footnotes). 
